# Purulent Arthritis

**Published:** 1882-07

**Authors:** William Henry Porter

**Affiliations:** Professor of Surgical Pathology and General Surgery


### ART. XVIII.—PURULENT ARTHRITIS: ONE OF A COURSE OF LECTURES DELIVERED AT THE COLUMBIA VETERINARY COLLEGE, N. Y. CITY, IN 1881-82.

BY WILLIAM HENRY PORTER, M.D., D.V.S.,
Professor of Surgical Pathology and General Surgery.

This is a special form of disease, differing somewhat from the suppurative arthritis of rheumatism, or the suppurative arthritis of white swelling. In this special form we have an abundant formation of pus, the rapidity of which is out of all proportion to the other inflammatory symptoms. This form of arthritis is met with in connection with purulent infectious diseases, viz., puerperal fever, small-pox, glanders, etc.

Pathological changes: The synovial membranes and fringes are congested, and changes in the cartilages can often be detected by the unaided eye. The prominent lesion, however, is the rapid formation of a large quantity of pus. The production of pus in this disease cannot be accounted for by a simple proliferation of endothelial cells. Here the Cohnheim's theory helps to explain the rapid development of pus, namely, a rapid migration and multiplication of the blood corpuscles. But with this it is hard to comprehend, especially where several joints become affected simultaneously.

Occasionally cases are met with in which the whole cartilage has been destroyed. In such cases the cartilages evidently melt down, and help to form the pus. This form of arthritis is rare, but may be seen in connection with cases of glanders. Suppurating joints of pyæmia come under this heading. Such a condition is occasionally seen in the newly born and still less frequently in the utero.

*Causes.*—Necrosis of the ends of long bones, caries, tubercular masses, as grapes of horned cattle, injuries, punctured wounds, and all the poisonous diseases already named which tend to cause suppurative inflammation. A simple acute arthritis, if prolonged, will of necessity, if not speedily cured, become chronic and assume a purulent character.

*Symptoms:* are pain, heat, swelling, and a peculiar position of the joint.

The *pain* is often severe, tense and burning; the movements of the building or the slightest jar increases the suffering, and any attempt at physical examination is followed by like results. The pain is universally greater at night.

The *heat* of the joint is considerably above that of the normal. There is often superficial redness.

The *swelling* in these cases is uniform, involving the whole articulation, and not projecting at single points, as in simple synovitis.

As a rule, the swelling is not elastic, but soft and doughy, and not fluctuating. The lack of positive fluctuation may be accounted for by the involvement of the surrounding tissues, which are also very much swollen and œdematous. As the disease advances, the swelling often increases suddenly and to a considerable extent, which increase may be the result of one of two things. First, there may be an increased irritation of the synovial membrane, which causes the production and accumulation suddenly of a large quantity of pus in the joint cavity. Second, the production of pus may be so great in quantity that the synovial sac gives way from over distention, and the pus is diffused into the surrounding cellular and muscular tissues, causing a diffuse suppurative cellulitis, thus causing an enormous and alarming swelling.

The *position* of the limb assumes that of renuflexion and eversion. In animals the tendency will be to rest only the toe on the ground.

*Spasms* or *twitching pains* of the limb often develop just as the patient is on the point of dropping to sleep, and this condition alone may deprive the patient of a night's sleep or several in succession.

The *constitutional* symptoms in a severe case are a high fever, pulse quick and irregular, a partial sweating of the whole body, with marked sweating of the affected limb. If the disease continues to advance and suppuration is fully developed in the joint, there is an increased heat in the joint accompanied by throbbing pain, and finally fluctuation may be detected. Occasionally in man the accumulation of pus is so rapid that laxation of the joint has occurred, but in equine surgery this would probably never be the result. Loosening and stretching of the ligaments occur, allowing the bones to grate against each other, when the articular cartilage has been destroyed and the ends of the bones laid bare. In other cases no grating is met with, sufficient cartilage being left at points to prevent the bared bones from coming together, or the surface of the denuded bone may be covered with a paste-like material which prevents the signs of grating. Cases are met with where there is every evidence of extensive erosion of the cartilages and yet no suppuration occurs. All the symptoms subside and recovery follows with but slight anchylosis. A nearly reverse condition is also true. Extensive suppuration occurs, but there is no laxation of the joint, no grating of the bones, but recovery is slow, and complete anchylosis the final result. In some of the cases where the capsule gives way, the pus escapes and is diffused widely throughout the tissues, or it gets between the periosteum and the bone great swelling and œdema of the limb will follow and we have incomplete fluctuation over a large territory. In such a case the swelling of the joint may completely subside and lead one to suppose that the trouble is past, but if the tissues are compressed above, the joint will speedily refill. In these cases the pus often makes its exit at some distance from the joint involved, a hectic condition often develops, and death instead of recovery is the result. The only condition which this form of joint affection could be mistaken for, would be an abscess forming close to and over a joint.

| *Joint Affection.* | *Abscess.* |
|---|---|
| Severe constitutional symptoms. | Less severe. |
| Great general swelling all around joint. | Less general swelling, marked at localized point. |
| Deep fluctuation. | Superficial fluctuation. |
| Rigidity of joint. | Absence of rigidity. |
| Preternatural mobility. | Mobility nearly normal. |
| Pain greater. | Pain less. |
| Spasms and startlings. | None. |
| Sometimes luxation. | No luxation. |
| Grating of the bones. | Never. |

The *treatment* is very easy to describe, but, like many things in equine surgery, exceedingly hard to put in practice fully and faithfully. The leading and principal thing is to secure perfect rest. Here again the plaster-of-Paris or Tripolith splint with extension ought to be used. The only possible way in which this can be accomplished is by raising the animal from the ground, in slings with extension, by a weight attached to the foot. When in this position, the apparatus should be applied, and when thoroughly set the limb will be immovable. The object in extension is to separate the articular surface and to secure perfect rest to the inflamed joint. Success in accomplishing these two things will give the best possible results in this, as in all other forms of joint affections. Warm fomentations, anæsthetic liniments, blisters, firing, etc., are recommended. Firing and blisters act more by increasing the pain and compelling the animal to keep the joint quiet than in any other way, and is a cruel method of treatment.

The shoe may be removed or a high-heeled one put on. The latter will give the animal the greater relief, but in case of recovery with anchylosis the limb will be practically worthless.

Mild cathartics should be used, and opium given to relieve the pain, guarding carefully against the constipation which is so apt to follow the constant use of opium.

